# Effects of HIV infection and ART on phenotype and function of circulating monocytes, natural killer, and innate lymphoid cells

**DOI:** 10.1186/s12981-018-0194-y

**Published:** 2018-03-15

**Authors:** Rose Nabatanzi, Stephen Cose, Moses Joloba, Sarah Rowland Jones, Damalie Nakanjako

**Affiliations:** 1 Department of Immunology and Molecular Biology, Makerere University College of Health Sciences, P. O. Box 7072, Kampala, Uganda; 2 MRC/UVRI Uganda Research Unit on AIDS and London School of Hygiene & Tropical Medicine, London, UK; 3 Nuffield Department of Medicine, University of Oxford, Oxford, UK; 4 Department of Medicine, Makerere University College of Health Sciences, Kampala, Uganda; 5 Infectious Diseases Institute, Makerere University College of Health Sciences, Kampala, Uganda

**Keywords:** HIV, Innate immunity, Monocytes, Natural killer cells, Innate lymphoid cells, Antiretroviral therapy

## Abstract

HIV infection causes upregulation of markers of inflammation, immune activation and apoptosis of host adaptive, and innate immune cells particularly monocytes, natural killer (NK) and innate lymphoid cells (ILCs). Although antiretroviral therapy (ART) restores CD4 T-cell counts, the persistent aberrant activation of monocytes, NK and ILCs observed likely contributes to the incomplete recovery of T-cell effector functions. A better understanding of the effects of HIV infection and ART on the phenotype and function of circulating monocytes, NK, and ILCs is required to guide development of novel therapeutic interventions to optimize immune recovery.

## Background

The human innate immune system is comprised of a complex network of cellular and soluble proteins that work together to provide the first-line of defense against common invading pathogens prior to involvement of the adaptive immune response [1–3]. Innate immune cells including monocytes, natural killer cells (NK), innate lymphoid cells (ILCs), and other antigen presenting cells (APCs) play a crucial role in the ushering in the adaptive arm of the immune response [4, 5]. In particular, monocytes are precursor cells to professional APCs involved in immune surveillance [6]. In addition, they have pattern-recognition receptors (PRRs) that detect conserved pathogen-associated molecular patterns (PAMPs) which lead to the induction of inflammatory responses that combat invading pathogens [7]. Natural killer cells produce cytokines; particularly interferon-gamma (IFN-ɣ) which activates phagocytic cells and primes APCs for interleukin 2 (IL-2) secretion thus shaping adaptive immunity towards a T helper 1 (Th1) response [8, 9]. ILCs rapidly secrete immunoregulatory cytokines which makes them provide protective immunity early on during infection [10] and also maintain intestinal homeostasis by directly regulating T cells through the presentation of peptide antigens on major histocompatibility complex II [11].

During HIV infection, the adaptive immune system is directly affected through the rapid infection of CD4 T-cells [12] but the effects on the innate immune system are more indirect through microbial translocation, inflammation, and immune activation [13]. Immune activation and inflammation cause a reduction in the numbers of monocytes, NK and ILCs, consequently leading to poor innate and adaptive immune responses, all which result in suboptimal response to infecting antigens [14].

Antiretroviral therapy (ART) suppresses HIV replication, restores CD4 T-cell numbers, reduces microbial translocation, inflammation, and aberrant T-cell activation [15–17]. The net effect of this is the near restoration of the immune system to pre-infection status and control/prevention of opportunistic infections and other AIDS-associated ailments [18, 19]. Several studies have however demonstrated incomplete recovery of the adaptive immune responses including ours which showed lower CD4 T-cell proliferation among ART-treated adults (with CD4 counts restored to 500 cells/µl and more), relative to their HIV-negative counterparts [19–21]. Innate immune cells; in particular NK cells, ILCs and monocytes, participate in the initiation and development of adaptive immune responses although little is known about their recovery during ART. This review discusses the recovery of monocytes, NK and ILCs during ART, because of their respective contributions to the regulation of the adaptive immune response.

**Fig. 1:**
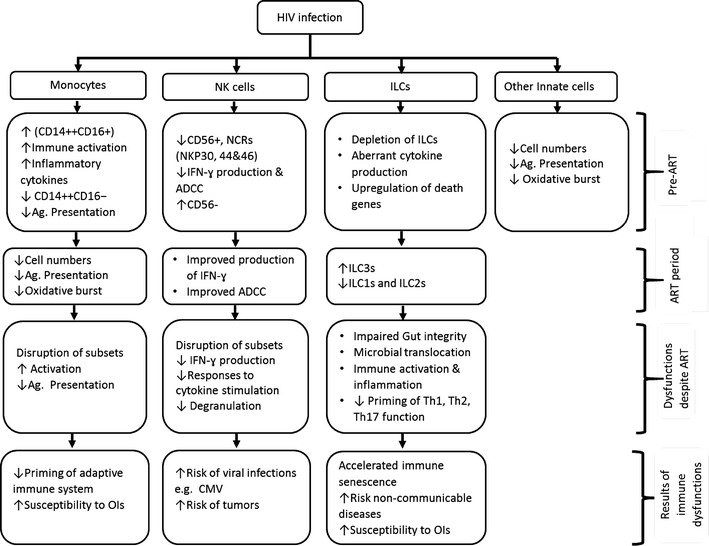
The effects of HIV infection and ART on monocytes, NK cells, ILCs

Figure 1 summarises the effects of HIV infection and ART on monocytes, NK cells, ILCs, and other innate cells. Persistent inflammation and activation of monocytes, NK cells, and ILCs is likely associated with the persistent T-cell activation and impaired effector functions observed among ART-treated adults [20–22]. There is likely a unique phenomenon of innate immune cell recovery during ART, among residents in sub-Saharan Africa (SSA) where several endemic infections activate the immune systems. A better understanding of innate cell dysfunctions and their effects on the adaptive responses during ART would guide the development of innovative therapeutic intervention to optimize recovery of host immune responses.

### HIV infection and innate immune cells

#### Monocytes

In the first few weeks of HIV infection, there is a massive accumulation of CD8 T-cells and a massive depletion of CD4 T-cells in the gut, followed by increased gut permeability and translocation of microbial products into circulation [23, 24]. Microbial translocation contributes to increased monocyte activation as evidenced by the rapid shift in the circulating monocyte pool from the classical phagocytic monocytes (CD14^++^CD16^−^) to the intermediate inflammatory monocyte subpopulation (CD14^++^CD16^+^) in the first 2 weeks of HIV infection [25]. Subsequently, monocyte subsets are disrupted, leading to suboptimal effector functions of phagocytosis, intracellular killing, chemotaxis and cytokine production [26]. HIV infection, both through direct infection and indirectly through microbial translocation, leads to monocyte activation and aberrant release of pro-inflammatory cytokines including TNF-α, IL-1β and IL-6, thereby activating the immune system [17]. In addition to aberrant cytokine production, HIV-associated monocyte activation leads to increased release of chemokines, leading to non-specific movement of monocytes into various tissue sites [23]. Direct HIV infection of the monocytes down regulates MHCII expression, inhibits MHCII-antigen complex formation and reduces the monocyte ability to take up antigens for processing and presentation to T cells [27]. In a study conducted in Beijing, Chen et al. [28] demonstrated that acute HIV-1 infected individuals had significantly increased proportions of inflammatory monocyte subsets and upregulated expression of the HLA-DR and CD163 receptors when compared with HIV negative individuals. These acquired defects in monocyte function cause the inability of monocytes to present antigens [27].

We postulate that the observed increase in inflammatory monocytes and immune activation markers could further impair monocyte responsiveness to antigens making the HIV infected individuals more susceptible to opportunistic infections. After several months to years of HIV infection, viral load levels gradually increase, while CD4 T-cells continue to reduce in number and function [29–31]. Similarly, there is dysregulation of monocyte subsets with higher populations of inflammatory (CD14^+^CD16^+^) monocytes than phagocytic (CD14^+^CD16^−^) populations. Monocytes in circulation become functionally anergic due to continued activation and high inflammatory status [32]. Untreated, individuals with chronic HIV-1 infection continue to have increased proportions of both the intermediate and non-classical monocytes subsets [33]. Moreover, levels of expression of CD163, a marker of activation on inflammatory monocytes remains significantly higher among individuals with chronic HIV-1-infection than HIV negative controls [34]. Protracted expression of the activated phenotype of monocyte subsets has a direct association with disease progression [33].

ART down regulates the excessive production of cytokines by the inflammatory monocytes thereby reducing the levels of immune activation and inflammation [17]. In a review by Burdo et al. [35], it was observed that ART initiation within the first year of HIV infection reduced monocyte activation, as evidenced by a reduction in expression of activation marker CD163 and absolute numbers of inflammatory monocytes. Similarly, markers of microbial translocation [lipopolysaccharide protein (LPS), IL-6, 16S ribosomal DNA and soluble CD14], and inflammatory markers such as d-dimer and interferon-α declined with ART initiation [33, 36]. Although a lot of immune functions appear to be recovered during ART, some monocyte dysfunctions persist. After 1 year of therapy, ART-treated adults were reported to still have elevated levels of the inflammatory monocyte subset (CD14^++^CD16^+^) and a downregulated expression of phagocytic monocyte subset (CD14^++^CD16^−^), resulting in the reduced ability of monocytes to process and present antigens to T cells [34, 37]. Similarly, phagocytic activity and oxidative burst of neutrophils and monocytes remained impaired among HIV-1 infected patients, in Athens general hospital after 3 months of ART [26, 38]. However, there is paucity of data on monocyte activation and functional recovery beyond 2 years of ART, particularly in sub-Saharan Africa (SSA) where monocyte frequency and functional recovery has not been widely studied in HIV treatment cohorts. Given the increasing numbers of individuals receiving ART, for 7 years and over, a better understanding of the effects of long-term ART on inflammation and monocyte activation would be relevant to inform innovations against chronic inflammation and its complications among adults living with HIV.

### Natural killer cells

Natural killer (NK) cells have an important role in controlling acute HIV infection, through rapid division and production of huge amounts of IFN-γ cytokine [8]. Strong NK cell activity and cytotoxic receptor expression are associated with preservation of CD4 T cells and lower viral set point [39]. HIV infection is associated with several changes in the NK cell compartment, including phenotypic and functional abnormalities that contribute to difficulty in the control of HIV progression [40]. Evidence of dysfunctional NK cell populations has been revealed by studies in the nonhuman primate model which have demonstrated anergic NK cell accumulation in lymph nodes in SIV infection [41].

In humans, acute HIV infection generally causes activation and expansion of the whole pool of NK cells [42], with abnormal distribution of the NK cell subsets. Pro-inflammatory NKCD56^bright^ populations are reduced, while the cytolytic CD56^dim^CD16^pos^ NK cell and dysfunctional CD56^neg^CD16^pos^ NK cells are increased in HIV positive people compared to HIV negative individuals [43, 44]. HIV infection reduces expression of the natural cytotoxicity receptors (NCR), NKp30, NKp44 and NKp46 [39, 45], which are essential in the containment and clearance of HIV virus. Evidence has further suggested that acute HIV infection activates the upregulation of stress ligands for cytotoxicity receptors including NKG2D which leads to lysis and cell death [46].

With continued viral replication, the CD56^dim^CD16^pos^ NK cell subset previously expanded in acute infection drop in numbers and function, as demonstrated by reduced CD107a expression and cytokine secretion [47]. The reduced numbers of cytolytic and cytokine producing NK cells would suggest that HIV infected patients with chronic disease remain susceptible to many infections especially those of viral origin [42, 48]. Evidence of reduced cytokine producing NK cells was further demonstrated in a rural Ugandan cohort, where chronically infected HIV individuals had lower expression of the NKG2A^+^CD57^+^CD56^dim^ subset in HIV infected group than the HIV negative controls [49]. In addition to down-regulated cytokine production, HIV infection causes a reduced ability of NK cells to perform ADCC due to a reduction in the number of the cytolytic CD56^dim^CD16^+^NK cells population [50] and a reduction in the intracellular stores of perforin and granzyme A [51].

Several discrepant results have been reported on the recovery of NK cells with ART.

ART has been shown to restore NK cell numbers with a mature phenotype in HIV-infected individuals, although defects of subset distribution and impaired ability to produce IFN-ɣ cytokine persist [44, 52, 53]. Frias et al. [54] reported incomplete recovery of NK cell subsets after 3 years of ART in Spain, in spite of undetectable viral load and an outstanding increase in the CD4 count to levels above 500 cell/µl. On the contrary, Mavilio et al. and Luo et al. [40, 55] reported complete recovery of NK cell subsets and functional profiles after 2 years of ART when compared with HIV negative individuals. In Ottawa at the immunodeficiency clinic, NK cell cytolytic activity was greatly reduced to levels similar to those among HIV negative people, after 1 year of ART [56]. In a Ugandan cohort, we demonstrated increased pro-inflammatory CD56^bright^ NK cells that were associated with suboptimal immune recovery despite 4 years of suppressive therapy [57]. Given that most of the evidence on the recovery of NK cells is from individuals after a short duration of ART, there is need to explore recovery of NK cell function after longer durations of ART. This is particularly important for sub-Saharan Africa which has more than 10 million people receiving ART majority of whom have been on treatment for more than 10 years [58].

### Innate lymphoid cells (ILCs)

Innate lymphoid cells are a group of innate immune cells that belong to the lymphoid lineage but do not respond in an antigen-specific manner, because of their lack of a B or T cell receptor [59]. These cells are subdivided into ILC1, ILC2 and ILC3 and these mirror the CD4 T helper cells TH1, TH2, and TH17 cells in the cytokines they produce. ILCs are mainly found at mucosal surfaces where they act as gatekeepers to invading infectious agents, including HIV [60]. ILCs rapidly secrete immunoregulatory cytokines which makes them provide protective immunity early on during infection [61]. Studies done in non-human primates revealed that ILC populations in the gut mucosa are significantly reduced in numbers due to an increase in cytotoxicity and inflammatory cytokine production by both ILCs and NK cells during acute Simian immunodeficiency viruses (SIV) infection. The reduction in ILC numbers contributes to the massive apoptosis and dysregulation in the gut-associated lymphoid tissue (GALT) [62, 63]. Xu et al. [64] showed that IL-17 producing ILC populations were drastically reduced in acute SIV infection, especially in the jejunum.

In humans, acute HIV infection has been associated with lymphoid tissue destruction of gut mucosa and further causes upregulation of genes associated with ILCs cell death, as evidenced by the depletion of ILCs both in blood and gut tissues of HIV-1 infected patients [63]. The destruction of lymphoid tissue cells has been associated with microbial translocation, immune activation and disease progression in both ART-treated and untreated individuals [65]. In a study by Kløverpris et al. among individuals with acute HIV infection in South Africa, it was demonstrated that all three subsets of ILCs were massively depleted from peripheral blood 7–14 days after HIV infection and these did not increase with viral load decreasing [65, 66].

In chronic HIV infection, ILC3s are further depleted and the depletion was attributed to the presence of excessive production of type 1 interferons by the plasmacytoid dendritic cells [67]. Although ILCs are depleted irreversibly from peripheral blood and the mucosal tissues, Mudd et al. [67] demonstrated that ILCs in tonsillar tissue are not significantly altered, meaning that ILC depletion is not generalised but rather compartmentalised and with continued therapy they may be redistributed back in peripheral blood and mucosal tissues.

Initiation of ART during acute HIV infection, preserves ILC numbers if it is initiated before peak viremia [66]. However, ART initiation during chronic HIV disease seems to have little effect on recovery of ILC numbers; with circulating ILC1s and ILC2s remaining significantly depleted and incomplete reconstitution of circulating ILC3s even with 2 years of ART [65, 66]. Kramer et al. studied ILC distribution in the gut and observed that despite effective use of ART, ILCs in HIV infected individuals remain dysregulated compared to their HIV negative counterparts. This lack of recovery of ILC distribution may contribute to the loss of intestinal barrier integrity and immune activation [66, 68, 69]. It is likely that individuals with persistent ILC dysfunction remain with limited mucosal protection and subsequently high risk of bacterial infections, autoimmune diseases and allergic infections due to the subsequent limitations in TH1, TH2 and TH17 functions that are mirrored by ILC1, ILC2 and ILC3 phenotypes, respectively [68].

### Consequences and clinical implications of persistent dysfunction of innate immune cells during ART

#### Innate immune cells and IRIS

ART generally leads to viral suppression, improvement of immune function, and better outcomes for many HIV positive individuals. Up to 10–25% of ART-treated individuals [70, 71] may develop the immune reconstitution inflammatory syndrome (IRIS) during the first months to years of HIV treatment. IRIS is a paradoxical inflammatory syndrome resulting from increased host immune responses to pre-existing opportunistic pathogens, including *Mycobacterium avium* complex, *M. tuberculosis*, *Cryptococcus neoformans*, Cytomegalovirus, JC virus, *Pneumocystis jirovecii*, Herpes zoster (VZV), and hepatitis B, as a result of CD4 T-cell restoration and interferon gamma production during the first months of ART [72]. The contribution of the different innate immune cells to IRIS has been documented by different groups; for example, in a Ugandan cohort, Tran et al. [73, 74] reported that monocyte-associated biological processes and functions were disturbed in TB-IRIS patients (after 2 weeks of ART), with dysregulation in both anti- and pro-inflammatory processes in monocytes. Andrade et al. [75] evaluated soluble biomarkers of inflammation and monocyte activation in patients who had been on ART for 12 weeks with TB-HIV co-infection from India and South Africa and found increased plasma levels of sCD14 and sCD163 pre vs post IRIS which are strong indicators of monocyte activation and predictors of death in TB-IRIS patients. Natural killer cells also exhibit phenotypic and functional differences in patients who develop IRIS relative to those who do not. At a Themba Lethu clinic Johannesburg, patients who developed IRIS had significantly higher levels of NK-cell degranulation before ART initiation [76] and exhibited high immune activation levels as demonstrated by the elevated levels of CD69 and HLA-DR [77]. Increased NK cell degranulation can cause lysis of cells infected with antigens, thereby increasing the circulating antigen load in these patients and contributing to the observed IRIS [76]. Given the propagating role of monocyte activation in IRIS processes, it is likely that therapeutic interventions to minimise monocyte activation might indirectly modify the risk and severity of IRIS among ART-treated adults.

#### Persistent immune activation and non-AIDS complications

Aberrant activation of the innate immune system is persistent despite ART [78], and it could be directly due to replicating HIV virus or indirectly through co-infections including subclinical Cytomegalovirus (CMV) infection [79]. Innate immune activation can be due to HIV directly infecting the monocytes/macrophages and dendritic cells or indirectly through HIV gene products like envelop proteins of gp120 and Nef that cause activation of lymphocytes and macrophages to produce pro-inflammatory cytokines and chemokines [80]. Evidence of indirect immune activation suggests that persistent leakage of lipopolysaccharide (LPS) into blood circulation [36] causes monocyte activation [81].

Persistent activation of innate immune cells is associated with the heightened production of pro-inflammatory cytokines (IL-1β, TNFα and IL-6) which cause T-cell activation. T-cell activation subsequently increases intracellular NF-κB levels which enhances the transcription of integrated virus and production of new virions that further infect more cells [82]. Activation of T-cells promotes T-cell depletion through upregulation of apoptosis, ADCC, and by-stander killing; all of which are functions of innate immune cells [3]. Reduced numbers of innate monocytes, NK and ILCs, consequently lead to poor innate and adaptive immune responses causing suboptimal response to infecting antigens [14]. Moreover, persistent inflammation and activation have been associated with fatal non-AIDS illnesses such as cardiovascular diseases, malignancies and organ damage among adults aging with HIV [83–85]. The high levels of inflammation and immune activation associated with chronic HIV disease, despite ART, contribute to accelerated immune aging and increase the risk of non-AIDS illnesses including cardiovascular diseases [86, 87], cataracts [88], malignancies [89, 90], bone demineralization [91], renal disease [92] and cognitive decline [93]) among HIV-positive adults relative to their HIV-negative counterparts. We, therefore, postulate that strategies to downgrade innate immune cell activation and associated dysfunctions could modify the magnitude, duration, and systemic complications of the aberrant immune activation associated with HIV chronic disease.

## Conclusions

HIV infection disrupts phenotypes and functions of monocytes, NK cells and ILCs, and subsequently the related adaptive host immune responses. ART restores some phenotypic and functional abnormalities associated with HIV infection, although persistent disruption of phenotypes and function of monocytes, NK cells, and innate lymphoid cells have been observed among populations of ART-treated adults. A further understanding of specific persistent innate immune cell phenotypic and functional abnormalities during ART is required to inform innovations in immune modulation interventions to optimize recovery of both innate and adaptive immune system. Similarly, a further understanding of the drivers of persistent immune activation is required to inform strategic therapeutic interventions to minimize its complications, particularly in sub-Saharan Africa where other infectious causes of immune activation such as malaria, tuberculosis and helminthic infections are still endemic.
